# Single-Entity Electrochemistry in the Agarose Hydrogel: Observation of Enhanced Signal Uniformity and Signal-to-Noise Ratio

**DOI:** 10.3390/gels9070537

**Published:** 2023-07-02

**Authors:** Jaedo Na, Kyungsoon Park, Seong Jung Kwon

**Affiliations:** 1 Department of Chemistry, Konkuk University, 120 Neungdong-ro, Gwangjin-gu, Seoul 05029, Republic of Korea; njd5458@konkuk.ac.kr; 2 Department of Chemistry and Cosmetics, Jeju Nation University, Jeju 63243, Republic of Korea

**Keywords:** singleientity electrochemistry, hydrogel, agarose, Pt nanoparticle, hydrazine oxidation

## Abstract

For the first time, single-entity electrochemistry (SEE) was demonstrated in a hydrogel matrix. SEE involves the investigation of the electrochemical characteristics of individual nanoparticles (NPs) by observing the signal generated when a single NP, suspended in an aqueous solution, collides with an electrode and triggers catalytic reactions. Challenges associated with SEE in electrolyte-containing solutions such as signal variation due to NP aggregation and noise fluctuation caused by convection phenomena can be addressed by employing a hydrogel matrix. The polymeric hydrogel matrix acts as a molecular sieve, effectively filtering out unexpected signals generated by aggregated NPs, resulting in more uniform signal observations compared to the case in a solution. Additionally, the hydrogel environment can reduce the background current fluctuations caused by natural convection and other factors such as impurities, facilitating easier signal analysis. Specifically, we performed SEE of platinum (Pt) NPs for hydrazine oxidation within the agarose hydrogel to observe the electrocatalytic reaction at a single NP level. The consistent porous structure of the agarose hydrogel leads to differential diffusion rates between individual NPs and reactants, resulting in variations in signal magnitude, shape, and frequency. The changes in the signal were analyzed in response to gel concentration variations.

## 1. Introduction

In recent years, single-entity electrochemistry (SEE) has gained significant attention as a powerful approach to studying the characteristics of nanomaterials at the individual level, rather than averaged properties as ensembles [1,2,3,4,5,6]. SEE has found application in various fields, including the investigation of electrocatalytic properties or mechanisms of metal [7,8,9] and metal oxide [10,11] nanoparticles (NPs), studies on soft NPs like emulsions [12,13], and examination of single biomaterials such as enzymes or cells [14,15].

Among the various nanomaterials, metal NPs have been widely used in many research fields owing to their attractive properties, which include a large surface-to-volume ratio, chemical reactivity, and size-dependent optical and electronic properties [16,17,18]. These enhanced characteristics of NPs compared with bulk materials can be applied to various modern scientific applications, such as electronic devices, sensors, medicine, spectrometry (such as Raman scattering enhancers), and catalysts [19,20,21,22]. Among these applications of NPs, its electrocatalytic effect has received a tremendous amount of attention. Because electrocatalysis is an essential key to the improvement of energy conversion and energy storage (such as oxygen reduction reaction (ORR)/oxygen evolution reaction (OER)/hydrogen evolution reaction (HER)), batteries, chemical synthesis, and electrochemical sensing systems [23,24,25]. It has been shown that the electrochemical activities of NPs are greatly altered depending on their size, shape, composition, and orientation. Therefore, numerous research efforts have been made to understand the electrocatalytic properties of NPs at the level of individual NPs and optimize them for practical applications.

In the SEE method, an electrode applying a predetermined voltage is immersed in a solution containing nanomaterials such as NPs. The NPs diffuse within the solution and eventually collide with the electrode, leading to the observation of a signal triggered by a specific electrochemical reaction induced by the NPs. However, the small current generated by a single NP is often challenging to distinguish from noise signals. Therefore, to obtain distinguishable signals, in other words, to enhance the signal-to-noise (S/N) ratio, fast electrochemical reactions or small electrodes such as ultramicroelectrodes (UMEs) are commonly employed. Although use of the UMEs has significantly reduced noise levels, residual fluctuations in noise current ranging from a few to tens of pA still exist. These fluctuations can be attributed to various physical and electrical factors, including the natural convection of the electrolyte solution [26,27].

Hydrogel, a three-dimensional crosslinked network polymer, has the ability to absorb and retain a significant amount of water while maintaining its structural solidity. It consists of a hydrophilic polymer chain with a high water content, typically ranging from 90% to 99% [28]. Recently, hydrogel has emerged as a valuable material in electrochemical applications due to its distinctive properties, such as large water content [29], biocompatibility [30,31], tunable swelling behavior [32,33], and easy integration with electrodes and active components [34,35,36]. Hydrogels serve as a sensing platform in electrochemical sensors, enabling selective detection of analytes [37,38,39]. Additionally, they provide useful applications in energy storage devices as electrodes or separators, facilitating enhanced charge storage and rapid ion diffusion [40,41]. In the realm of electrochemical actuators, hydrogel acts as an artificial muscle, responding to electric potential and generating mechanical work [42,43]. Moreover, hydrogel is employed in electrocatalysis, providing a supporting matrix for electrocatalytic materials to enhance various reactions [44,45]. The hydrogel is applied not only in the electrochemical field, but also in various fields such as drug delivery system [46], mechanics [47,48], and environmental fields [49].

Recent studies by Park et al. have demonstrated that, when hydrogel is used as solid electrolytes in electrochemical experiments, the influence of convection can be reduced, simplifying the mass transfer of reactants [35,36]. This finding suggests that it holds potential for improving the S/N ratio in the SEE by mitigating noise from convection in electrochemical measurements and simplifying the mass transfer model.

If the SEE is performed in a hydrogel matrix instead of a conventional solution media, it can offer several advantages. Colloidal NPs may exhibit decreased stability in the electrolyte solution caused by the agglomeration of each particle [50]. Although stabilizing agents (capping agents) are used in the synthetic process of NP to control the growth and to prevent aggregation, excessive use of these capping agents can affect the catalytic ability of the NP surface. In addition, NPs are often agglomerated together in the electrolyte solution. In such cases, even with the use of uniform NPs, the magnitude of the signal, which is proportional to the size of the NPs, can be interrupted due to the NP’s aggregation. However, the hydrogel matrix acts as a molecular sieve, effectively filtering out the aggregated NPs. This results in a more uniform signal that accurately represents the size distribution of the NPs. Additionally, the gel environment minimizes background current fluctuations caused by other factors, such as impurities, thereby facilitating easier signal analysis and increasing the S/N ratio.

In this paper, for the first time, the SEE of a single NP was observed in a hydrogel matrix. We investigated the effects of hydrogel incorporation on the mass transfer of NPs in the context of SEE. By examining the changes in magnitude, shape, and frequency of SEE signals depending on the composition of the hydrogel matrix, we elucidated the potential of hydrogel-based SEE for enhanced signal analysis and improved understanding of individual nanomaterials.

## 2. Results and Discussion

The hydrogel can be considered as a network of cross-linked polymer chains which possess a wide-spreading “pore size” or “mesh size” ranging from 1 to 900 nm depending on the agarose concentration [51]. Such porous structures can affect the mass transfer rate of materials or cause additional overvoltage, which may affect the rate of electrochemical reactions. Thus, to validate the electrochemical behavior within the agarose gel and determine the optimal potential for single-entity electrochemistry (SEE), electrochemistry for hydrazine oxidation at various electrodes was investigated in the agarose hydrogel.

**Figure 1:**
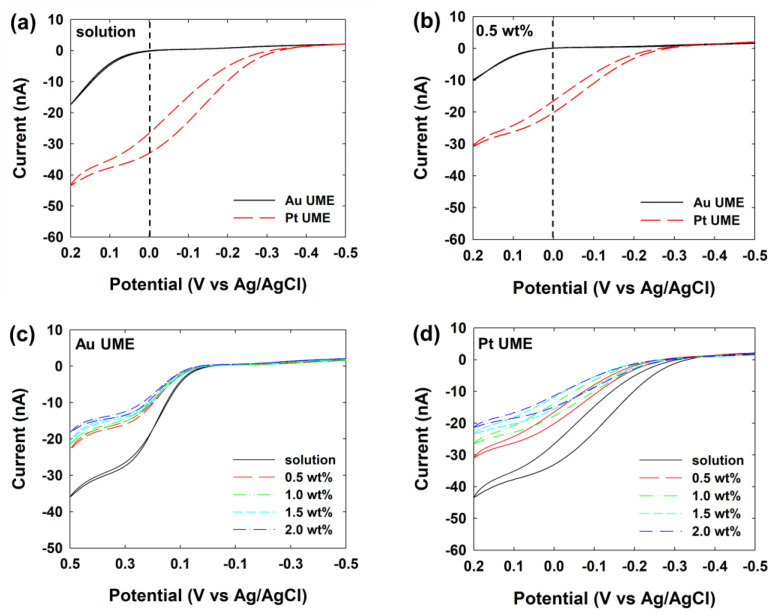
Cyclic voltammograms of the hydrazine oxidation using Au (black solid) and Pt (red dashed) UMEs in (**a**) solution or (**b**) 0.5 wt% agarose hydrogel. Cyclic voltammograms of the hydrazine oxidation using (**c**) Au and (**d**) Pt UMEs at various concentrations of the agarose hydrogel (from 0.5 to 2.0 wt%). Both electrolytes contain 50 mM PB (pH 7.0) and 5 mM hydrazine. The scan rate was 50 mV.

First of all, cyclic voltammetry (CV) experiments were conducted using Pt and Au ultramicroelectrodes (UMEs) in both aqueous solution and 0.5 wt% agarose hydrogel to assess the electrocatalytic properties depending on the electrode materials and diffusional properties between the two media (Figure 1a,b). The CV results revealed that the Pt UME exhibited a lower onset potential for hydrazine oxidation compared to the Au UME, irrespective of the medium employed. At a potential around 0 V (vs. Ag/AgCl), the Pt UME demonstrated an electrocatalytic current resulting from the hydrazine oxidation, while the Au UME did not. This difference in electrocatalytic behavior motivated the selection of this potential for subsequent SEE analyses. For SEE of metal NPs using electrocatalytic amplification, it should be noted that the appropriate electrode potential needs to be selected. This potential should give rise to the maximum current for the particle material, while the electrode exhibits a minimum current response at the given electrocatalytic reaction.

In terms of steady-state currents, a noticeable disparity was observed between the aqueous solution and the agarose hydrogel. While the onset potentials remained similar, the steady-state current levels in the agarose hydrogel were lower compared to those in the aqueous solution. The steady-state current at an UME is directly proportional to the diffusion of reactants to the electrode surface, suggesting reduction in the diffusion coefficient and subsequent decrease in current within the gel matrix. This observation was further supported by the gradual decline in steady-state current with increasing concentration of the agarose hydrogel (Figure 1c,d).

The steady-state current in the disk-type UME is obtained as follow [52,53]:

(1) $$I_{ss,UME}=4FD_{hyd}C_{hyd}r_{UME}$$

where *D_hyd_* is the diffusion coefficient of hydrazine, *C_hyd_* is the concentration of hydrazine, and *r_UME_* is the radius of UME. These results can be derived from the hindered mobility of the redox molecule in agarose gel, which is assumed to be caused by steric interaction between solutes and agarose gel fibers [54]. The diffusion coefficients of hydrazine, calculated from the steady-state current, were summarized in Table 1. Notably, the diffusion coefficient of hydrazine in the 0.5 wt% agarose hydrogel was lower (approximately 84%) than that of in aqueous solution.

In conclusion, it is considered possible to perform SEE in agarose gel due to significant differences in catalytic properties between Au and Pt despite a slight decrease in the diffusion coefficient observed in agarose gel.

**Scheme 1:**
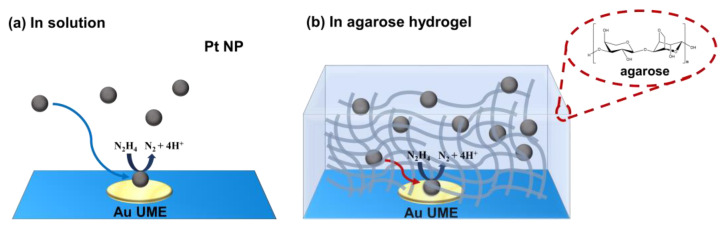
Schematic illustration of difference diffusion pathway of Pt NP in (**a**) aqueous solution (**b**) agarose hydrogel.

As depicted in Scheme 1, SEE of Pt NPs was conducted in both aqueous solution (liquid electrolyte) and agarose hydrogels (solid electrolyte). To enable the SEE within a hydrogel matrix, it is imperative that nanoparticles (NPs) can readily penetrate the hydrogel structure. The pore size of the prepared hydrogel is expected to range from tens to hundreds of nanometers, depending on the concentration, ranging from 2.0 to 0.5 wt%, as reported in the literature [51]. Therefore, it is thought that the ~50 nm of Pt NPs may or may not penetrate the hydrogel depending on the pore size of the hydrogel.

**Figure 2:**
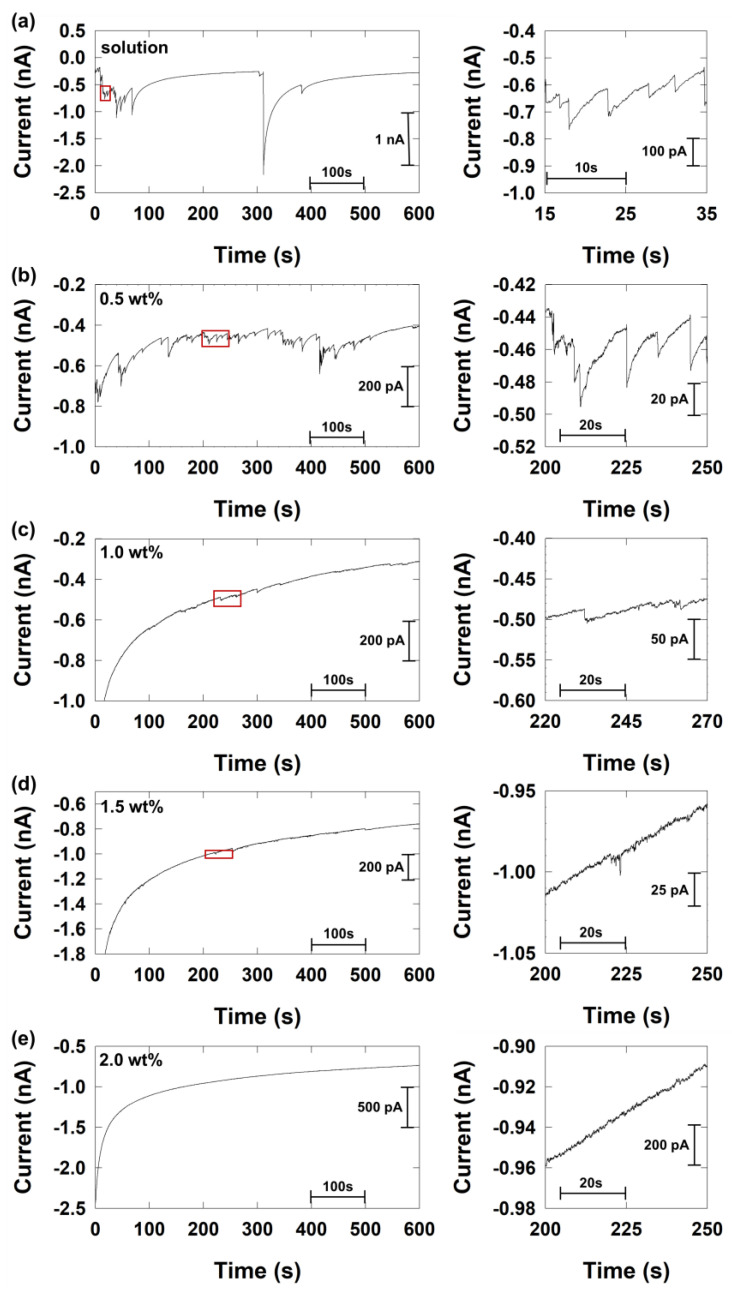
Chronoamperometric curves for single Pt NP collision at the Au UME with different concentrations of the agarose hydrogel (**a**) 0 wt% (solution) (**b**) 0.5 wt% (**c**) 1.0 wt% (**d**) 1.5 wt% (**e**) 2.0 wt%. All electrolyte solutions contained 50 mM PB (pH 7.0) and 5 mM hydrazine. The Pt NP concentration was 0.8 pM at solution, and 20 pM at agarose hydrogel. The data acquisition time was 50 ms.

The magnitude and frequency of the current signal of the SEE were investigated at various concentrations of agarose hydrogel, compared to the result in aqueous solution. (Figure 2). Owing to the slow diffusion within the agarose hydrogel compared to the solution, the agarose hydrogel pre-equilibrated with a high concentration of Pt NP solution and hydrazine was used for the SEE. Specifically, the Pt NP concentration was 0.8 pM in the solution and 20 pM in the agarose hydrogel.

As shown in Figure 2a and consistent with previous studies, the SEE of Pt NPs exhibited a staircase current signal in the aqueous solution. Each current step implies a steady state current generated by individual Pt nanoparticles collided onto the Au electrode. The magnitude of the current signal and collisional frequency were calculated using the equations of previous studies [52,53]:

(2) $$I_{ss,NP}=4\pi \left(ln2\right)nFD_{hyd}C_{hyd}r_{NP}$$

and

(3) $$f_{P}=4D_{NP}C_{NP}r_{UME}$$

where *r_NP_* is the radius of NP, the *D_NP_* is the diffusion coefficient of NP, and *C_NP_* is the concentration of NP.

The theoretical expectations and experimental observations of the signal magnitudes and frequencies are summarized in Table 2. The estimated values obtained from Equations (2) and (3) were 330 pA for the current step and 0.104 s^−1^ for the frequency in the liquid electrolyte. The experimentally obtained values were 495 (±338) pA and 0.028 s^−1^, respectively. The two values roughly coincide, but the experimentally observed data was slightly larger in current magnitude and lower in frequency, which is believed to be due to the occurrence of NP aggregation in the solution. It has been reported that the aggregation of Pt NPs in hydrazine solution [50]. Therefore, the aggregation can lead to inconsistent signal magnitudes and a large coefficient of variation (68%). As a result, when attempting to predict the real size distribution of NPs based on the magnitude of the current signal, accurate predictions are challenging due to signal distortion caused by such aggregation in an electrolyte solution.

In the 0.5 wt% agarose hydrogel (Figure 2b), a current step of 27 (±13) pA was observed. Considering only the decrease in the diffusion coefficient of hydrazine in the agarose hydrogel (84% from Table 1), the expected current magnitude in the solid electrolyte should be 278 pA. Hence, it is suspected that additional factors contribute to this phenomenon. Furthermore, triangular-shaped current responses were observed in the case of 0.5 wt% agarose hydrogel, which deviates from the staircase response in the solution electrolyte. This triangular shape implies that the mass transfer of hydrazine is diminishing and ultimately restricted due to some reason. We speculate that this limitation is due to the blocking effect of the N_2_ gas, which is a product of the oxidation reaction of hydrazine. The oxidation reaction of hydrazine can be represented as follows:

$$N_{2}H_{4}\to N_{2}+4H^{+}+4e^{-}$$

Therefore, the generated N_2_ gas has greater difficulty in escaping from the NP surface within the solid electrolyte compared to the liquid electrolyte. As a result, it is hypothesized that the N_2_ gas hinders the mass transfer of the reagent, leading to a decrease in the current signal.

The pore size of the prepared hydrogel may decrease as the concentration of hydrogel increases. As a result, the magnitude of the SEE signal was gradually reduced, and small signals resembling spike shapes were observed at 1.0 and 1.5% (Figure 2c,d). Finally, no observable signal was obtained at concentrations above 2.0 wt% (Figure 2e). Previous studies on the pore size of agarose gel show that when it reaches 2.0 wt%, the pore size decreases a lot compared to when it is 0.5 wt% (Table 3) [55,56,57,58]. Consequently, since the pore size of 2.0 wt% is similar to or smaller than the Pt NP size of 50 nm, the Pt NPs are difficult to move freely in the gel network, resulting in the disappearance of the signal.

**Figure 3:**
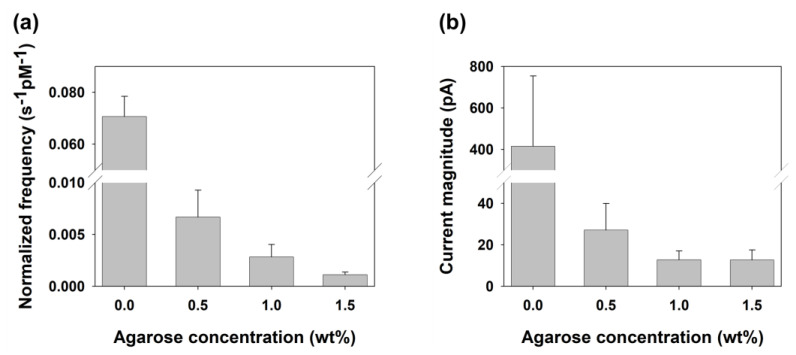
(**a**) Normalized collisional frequency and (**b**) current magnitude of the SEE signal as a function of agarose hydrogel concentration.

Furthermore, in the 0.5 wt% agarose hydrogel, the collision frequency decreased to less than 1/10 compared to that in the solution electrolyte. As the concentration of the agarose hydrogel increases, this reduction continues. This indicates that the diffusion of NPs is hindered within the agarose hydrogel, resulting in a slower rate of diffusion (Figure 3a)

Despite the reduction in current signal magnitude due to the decreased diffusion coefficient, the uniformity of signals was relatively improved in the agarose hydrogel since NP aggregation was less pronounced in the agarose hydrogel.

The appearance of aggregation results in the formation of larger NPs, and the average particle size increases while the NP concentration decreases. Consequently, an increase in the average signal magnitude and a decrease in collisional frequency in SEE are observed. As listed in Table 4, in the aqueous solution, the current magnitude and frequency during the 200–400 s time range decreased and increased from 330 to 1050 and from 0.075 to 0.015, respectively, compared to the values in the 0–200 s time range. This observation aligns with the expected effect of the NP aggregation mentioned earlier. On the other hand, in the 0.5 wt% agarose gel, there was no significant change in the current magnitude and frequency between the 200–400 s and 0–200 s time range.

Therefore, the uniformity of the current signal can be assessed by the coefficient of variation of the current magnitude, which increased to 47% in the agarose hydrogel compared to 68% in the solution (Figure 3b). In this regard, it is noteworthy that the entangled network fibers of the hydrogel not only affect the diffusion properties but also serve as a molecular body that prevents NPs aggregation inside the hydrogel.

In the agarose hydrogel environment, mass transport is reduced, especially that due to convection, and background current fluctuations caused by factors such as impurities are minimized, leading to a decrease in the magnitude of noise. As shown in Table 5, the actual magnitude of noise significantly decreased in the agarose hydrogel, resulting in an enhanced signal-to-noise (S/N) ratio. The S/N ratio was calculated by dividing the magnitude of the current response by the background current fluctuation. The improved S/N ratio can benefit the detection of NPs, especially small ones with low signal levels.

## 3. Conclusions

For the first time, the single-entity electrochemistry (SEE) of Pt nanoparticles (NPs) was conducted in a solid electrolyte, such as agarose hydrogel. In the 0.5 wt% agarose hydrogel, the diffusion of NPs and reagent was slower compared to that in the liquid electrolyte, resulting in a decrease in signal magnitude and frequency. However, it can offer several advantages. The agarose hydrogel matrix excludes signals generated by aggregated NPs, resulting in more uniform signals. By minimizing background current fluctuations caused by factors like natural convection and impurities, the signal-to-noise (S/N) ratio is increased, making signal analysis more convenient. These change in current response was investigated as a function of agarose hydrogel concentration.

This study demonstrates that the improved S/N ratio and uniformity of signal in solid electrolytes can be utilized for the observation of SEE of NPs with small signal magnitudes, which are difficult to observe in liquid electrolytes. It also provides inspiration for future investigations into the electrochemical behavior of NPs in various solid electrolytes such as a hydrogel.

## 4. Materials and Methods

### 4.1. Reagents

All chemicals used as received. Agarose (Low Electroendosmosis), platinum nanosphere (Pt NP, 50 nm), hydrazine (N_2_H_4_), potassium phosphate monobasic (KH_2_PO_4_), potassium phosphate dibasic (K_2_HPO_4_) were obtained from Sigma-Aldrich (St. Louis, MO, USA). Ultrapure water (>18 MΩ, Millipore, Darmstadt, Germany) was used in all experiments. Gold (Au, 99.99%, diameter 10 μm) and Pt (99.99%, diameter 10 μm) were purchased from Alfa Aesar (Ward Hill, MA, USA).

### 4.2. Characterization of Pt NPs

**Figure 4:**
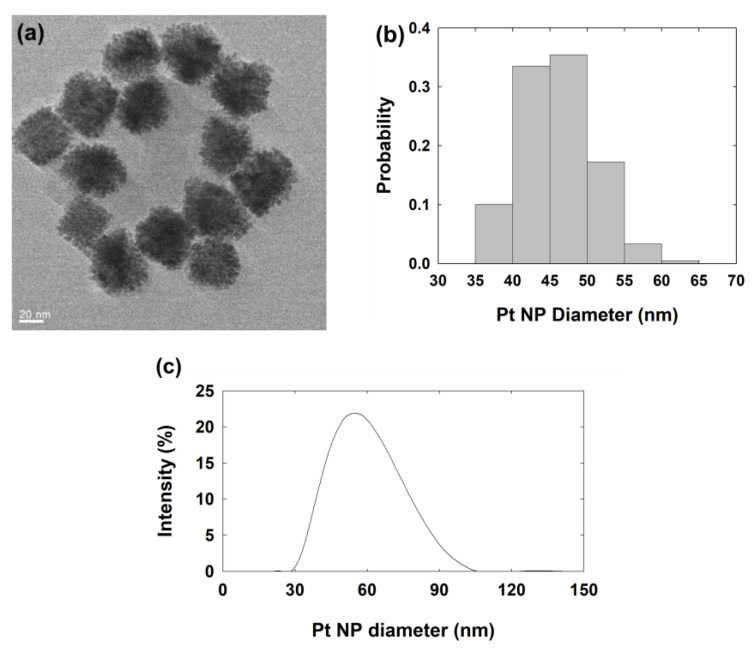
(**a**) Transmission Electron Microscopy (TEM) image, (**b**) size distribution of Pt NP measured with TEM, (**c**) size distribution of Pt NP measured with Dynamic Light Scattering (DLS) analysis. The average diameter (±standard deviation) is 45 (±5) nm with TEM and 57 (±14) nm with DLS.

The size of the Pt NPs was characterized using Transmission Electron Microscopy (TEM) images and dynamic light scattering (DLS) analysis. As shown in Figure 4, the analyzed metal part size of Pt NP by TEM was 45 (±5) nm and the hydrodynamic diameter of Pt NP by DLS was 57 (±14) nm in diameter. The diameter from the DLS analysis was larger than that measured from the TEM images. This difference seems to be due to the hydrodynamic environment during DLS measurements and the dry environment at TEM measurements. The concentration of Pt nanoparticles (NPs) was estimated by dividing the concentration of the Pt precursor by the number of Pt atoms present in the average size of NP from the TEM image. The estimated concentration of the stock solution of Pt NP was 80 pM.

### 4.3. Preparation of Agarose Hydrogel

Various concentrations (0.5, 1.0, 1.5, and 2.0 wt% in water) of agarose solutions were prepared in a flask connected to the reflux tube. Agarose solutions were heated up to 90 °C for complete dissolution. The agarose solutions were put into a vacuum chamber to remove air bubbles. After, the solution was placed in a silicone mold and slowly cooled to room temperature to harden it. The hardened gel was cut into 1 cm cubes and stored in distilled water. The pore size of agarose hydrogel can be measured in the various methods previously studied such as DNA mobilities [55], absorbance change [56], surface fraction change [57], and scanning electron cryo-microscopy (Cryo-SEM) [58]. According to these methods, The pore diameter of 0.5~2.0 wt% agarose hydrogel can be estimated to be 250~40 nm [55], 450~40 nm [56], 280~6 nm [57], or 330~150 nm [58], as listed in Table 3.

**Figure 5:**
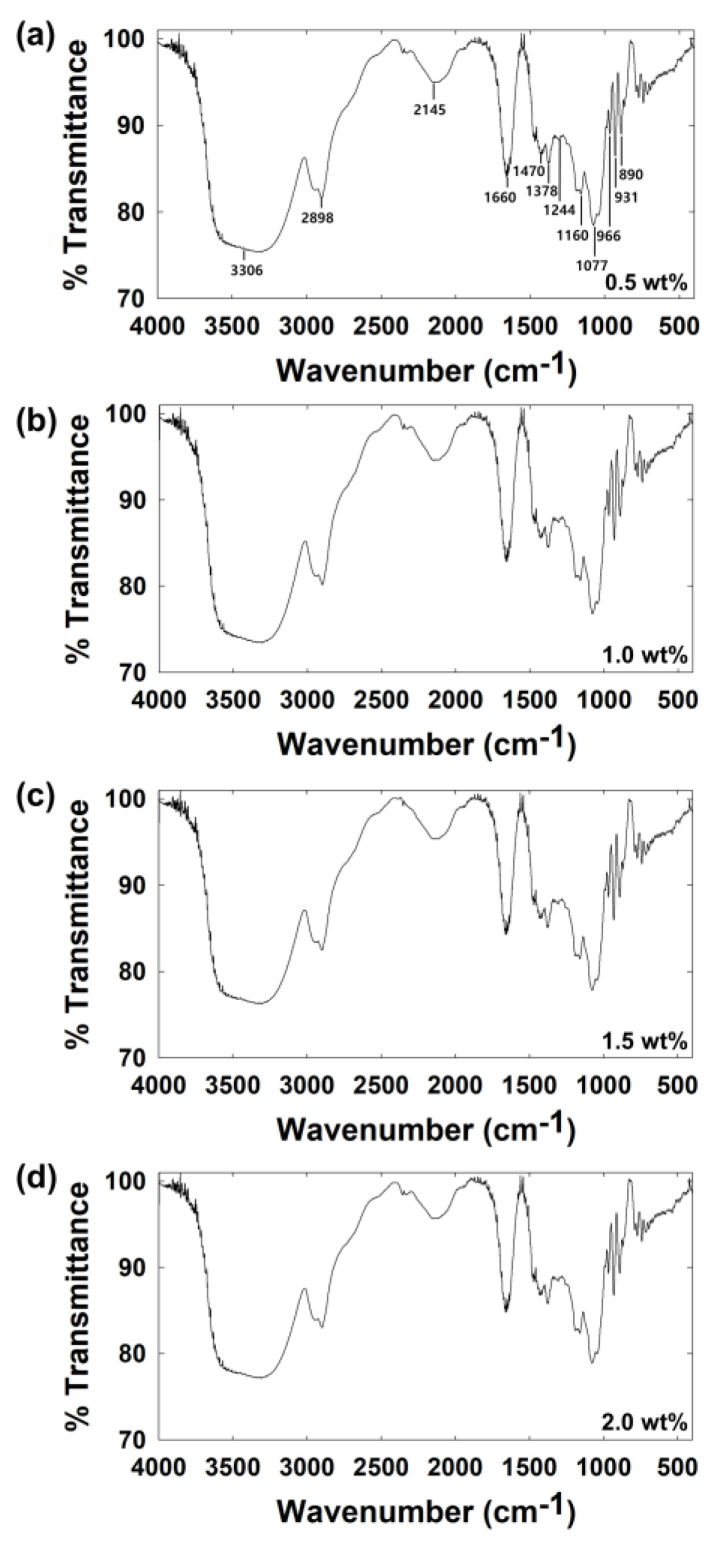
FT-IR spectra of various concentration of the agarose hydrogel with (**a**) 0.5 wt%, (**b**) 1.0 wt%, (**c**) 1.5 wt%, and (**d**) 2.0 wt%.

Fourier transform infrared (FT-IR) measurements were conducted at various concentrations of agarose hydrogel (Figure 5). A thinly sliced and dried agarose hydrogel was used as a sample for measurement. As shown in Figure 5, peaks were obtained at the same location regardless of agarose concentration. The broad absorption band at 3306 cm^−1^ was attributed to the stretching vibration mode of -OH groups. The band at 2898 cm^−1^ was related to the stretching vibration mode of -CH_2_ groups. The band at 1660 cm^−1^ was attributed to the stretching vibration of the bound H_2_O. Peaks at 1470 cm^−1^ and 1378 cm^−1^ were related to the bending vibration of the -CH_2_ and -CH_3_ groups, respectively. The vibration of the covalent sulfate group is indicated by the weak band at 1244 cm^−1^. Broad bands at 1160 cm^−1^ and 1077 cm^−1^ were related to the vibration of C-O-C groups about the glycosidic linkage. These peaks correspond with the known peaks for agarose gel [59,60].

**Figure 6:**
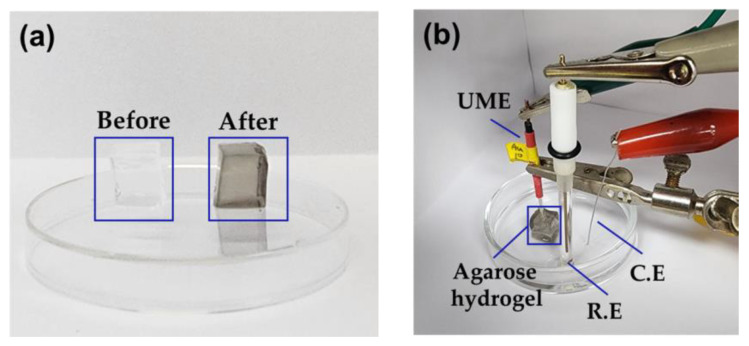
(**a**) 0.5 wt% agarose hydrogels before (left, white) and after (right, black) equilibrium with Pt NPs and hydrazine solution. (**b**) electrochemical cell setup with hydrogel.

For electrochemical measurement, a cubic agarose hydrogel was soaked in 1 mL of Pt NP stock solution for 72 h until the Pt NPs were reached in equilibrium between the agarose hydrogel and solution. The concentration of Pt NPs in agarose hydrogel was estimated at ~40 pM, which is half the concentration of the stock solution. Then, the agarose hydrogel containing Pt NPs was immersed in 1 mL 100 mM phosphate buffer (PB) solution containing 10 mM hydrazine for 8 h. After two equilibrium steps, for example, the 0.5 wt% agarose gel is considered to contain ~20 pM Pt NP, ~5 mM hydrazine, and 50 mM PB [37,38]. The hydrogels before and after equilibrium with Pt NPs and hydrazine solution are shown in Figure 6a.

### 4.4. Preparation of UMEs

UMEs were fabricated as previously reported [7,10,61]. First, one side of the borosilicate glass tube was sealed with a torch. A 10 µm diameter of metal (Au or Pt) wire was put into a sealed borosilicate glass. The opposite side of the glass tube was connected to the vacuum pump. The sealed side of the glass tube was sealed using nichrome wire under vacuum. Next, the remained metal wire was connected to the lead line using silver epoxy. The sealed glass was ground with sandpaper to expose the metal surface, and polished with alumina powder for the mirror surface of the electrode.

### 4.5. Instruments

The electrochemical experiments were performed by using a CHI 750e potentiostat (CH Instruments, Austin, TX, USA) with three electrode cells placed in a Faraday cage. The three-electrode system consisted of UME as a working electrode, Pt wire as a counter electrode, and Ag/AgCl 1 M KCl as a reference electrode. TEM images were obtained using a JEM-3010 (JEOL Ltd., Tokyo, Japan). DLS analysis was performed using a Zetasizer Nano ZS90 (Malvern, Worcestershire, UK). FT-IR was preformed using a Nicolet 6700 FT-IR spectrometer (Thermo Fisher Scientific, Waltham, MA, USA).

### 4.6. Electrochemical Cell

All electrochemical measurements were performed in a three-electrode cell system. A 50 mM phosphate buffer (PB) solution containing 5 mM hydrazine was used for an electrolyte solution. Agarose hydrogel was placed in electrolyte solution and the working electrode connected to the agarose hydrogel. Pt counter electrode and Ag/AgCl 1 M KCl reference electrode were placed outside agarose hydrogel in solution as shown in Figure 6b.

When the measurement in 0 wt% agarose hydrogel, we used 50 mM PB containing 5 mM hydrazine solution for the electrolyte, and all three electrodes were placed in the electrolyte solution without agarose gel, and the Pt NPs were injected into the solution after chronoamperometric measurement had started.

## Figures and Tables

**Table 1:** Calculated diffusion coefficient of hydrazine at various concentrations of agarose hydrogel.

Concentration of Agarose Hydrogel (wt%)	Steady-State Current (nA)	Calculated Diffusion Coefficient of Hydrazine ^a^ (×10^−6^ cm^2^/s)
0 (solution)	33.8	8.75
0.5	28.4	7.37
1.0	25.5	6.59
1.5	20.3	5.25
2.0	18.5	4.79

^a^ Calculated by Equation (1).

**Table 2:** Current magnitude and frequency of SEE signal.

Concentration of Agarose Hydrogel (wt%)	Calculated Current Magnitude ^a^ (pA)	Experimental Current Magnitude (pA) (±SD)	Calculated Frequency ^b^ (s^−1^)	Experimental Frequency (s^−1^) (±SD)	Normalized Frequency (s^−1^·pM^−1^) (±SD)
0 (solution)	330	495 (±338)	0.104	0.028 (±0.003) ^c^	0.035 (±0.004)
0.5	278	27 (±13)	-	0.066 (±0.026) ^d^	0.003 (±0.001)
1.0	249	13 (±4)	-	0.028 (±0.012) ^d^	0.001 (±0.001)
1.5	198	12 (±5)	-	0.011 (±0.003) ^d^	0.0005 (±0.0001)
2.0	181	-	-	0 ^d^	0

^a^ Calculated by Equation (2); ^b^ Calculated by Equation (3); ^c^ Observed at 0.8 pM of Pt NP; ^d^ Observed at 20 pM of Pt NP; SD: standard deviation.

**Table 3:** Previous studies of pore size of agarose hydrogel as a function of concentration.

Method Based on	Pore Diameter (nm)				Reference
	0.5 wt%	1.0 wt%	1.5 wt%	2.0 wt%	
DNA mobilities change	250	100	60	40	[55]
absorbance change	450	150	70	45	[56]
surface fraction change	280	85	71	6	[57]
Cryo-SEM	330	230	190	150	[58]

**Table 4:** Average current magnitude and frequency of SEE signal at every 200 s range.

Time Range (s)	0 wt% (Solution)		0.5 wt%	
	Experimental Current Magnitude (pA) (±SD)	Experimental Frequency (s^−1^)	Experimental Current Magnitude (pA) (±SD)	Experimental Frequency (s^−1^)
0~200	330 (±180)	0.075	31.8 (±15)	0.13
200–400	1050 (±1140)	0.015	32.2 (±18)	0.12

**Table 5:** Comparison of the signal-to-noise ratio at various concentrations of agarose hydrogel.

Concentration of Agarose Hydrogel (wt%)	Experimental Current Magnitude (pA)	Noise Level ^a^ (pA)	Signal to Noise Ratio (S/N Ratio)
0 (solution)	495	53	7.8
0.5	27	0.65	42
1.0	13	0.79	16
1.5	12	0.85	15

^a^ Estimated from the background current fluctuation.

## Data Availability

Not applicable.

## References

[1] Stark W.J., Stoessel P.R., Wohlleben W., Hafner A.J.C.S.R. (2015). Industrial applications of nanoparticles. Chem. Soc. Rev..

[2] Van Broekhuizen P., van Broekhuizen F., Cornelissen R., Reijnders L. (2011). Use of nanomaterials in the European construction industry and some occupational health aspects thereof. J. Nanopart. Res..

[3] Bauer L.A., Birenbaum N.S., Meyer G.J. (2004). Biological applications of high aspect ratio nanoparticles. J. Mater. Chem..

[4] Liebel M., Calderon I., Pazos-Perez N., van Hulst N.F., Alvarez-Puebla R.A. (2022). Widefield SERS for High-Throughput Nanoparticle Screening. Angew. Chem. Int. Ed..

[5] Chen J., Lim B., Lee E.P., Xia Y. (2009). Shape-controlled synthesis of platinum nanocrystals for catalytic and electrocatalytic applications. Nano Today.

[6] Spendelow J.S., Wieckowski A. (2007). Electrocatalysis of oxygen reduction and small alcohol oxidation in alkaline media. Phys. Chem. Chem. Phys..

[7] Xiao X., Bard A.J. (2007). Observing single nanoparticle collisions at an ultramicroelectrode by electrocatalytic amplification. J. Am. Chem. Soc..

[8] Zhou Y.G., Rees N.V., Compton R.G. (2011). The electrochemical detection and characterization of silver nanoparticles in aqueous solution. Angew. Chem. Int. Ed..

[9] Zhou H., Fan F.R.F., Bard A.J. (2010). Observation of discrete au nanoparticle collisions by electrocatalytic amplification using Pt ultramicroelectrode surface modification. J. Phys. Chem. Lett..

[10] Kwon S.J., Fan F.R.F., Bard A.J. (2010). Observing iridium oxide (IrOx) single nanoparticle collisions at ultramicroelectrodes. J. Am. Chem. Soc..

[11] Fernando A., Parajuli F., Alpuche-Aviles M.A. (2013). Observation of individual semiconducting nanoparticle collisions by stochastic photoelectrochemical currents. J. Am. Chem. Soc..

[12] Kim B.K., Kim J., Bard A.J. (2015). Electrochemistry of a single attoliter emulsion droplet in collisions. J. Am. Chem. Soc..

[13] Kim B.K., Boika A., Kim J., Dick J.E., Bard A.J. (2014). Characterizing emulsions by observation of single droplet collisions—Attoliter electrochemical reactors. J. Am. Chem. Soc..

[14] Dick J.E., Hilterbrand A.T., Boika A., Upton J.W., Bard A.J. (2015). Electrochemical detection of a single cytomegalovirus at an ultramicroelectrode and its antibody anchoring. Proc. Natl. Acad. Sci. USA.

[15] Dick J.E., Renault C., Bard A.J. (2015). Observation of single-protein and DNA macromolecule collisions on ultramicroelectrodes. J. Am. Chem. Soc..

[16] Emory S.R., Haskins W.E., Nie S. (1998). Direct observation of size-dependent optical enhancement in single metal nanoparticles. J. Am. Chem. Soc..

[17] Balamurugan B., Maruyama T. (2005). Evidence of an enhanced interband absorption in Au nanoparticles: Size-dependent electronic structure and optical properties. Appl. Phys. Lett..

[18] Shankar S.S., Rai A., Ankamwar B., Singh A., Ahmad A., Sastry M. (2004). Biological synthesis of triangular gold nanoprisms. Nat. Mater..

[19] Wei H., Hao F., Huang Y., Wang W., Nordlander P., Xu H. (2008). Polarization dependence of surface-enhanced Raman scattering in gold nanoparticle−nanowire systems. Nano Lett..

[20] Wilczewska A.Z., Niemirowicz K., Markiewicz K.H., Car H. (2012). Nanoparticles as drug delivery systems. Pharmacol. Res..

[21] Wang C., Yu C. (2013). Detection of chemical pollutants in water using gold nanoparticles as sensors: A review. Rev. Anal. Chem..

[22] Yahya R., Shah A., Kokab T., Ullah N., Hakeem M.K., Hayat M., Shah I. (2022). Electrochemical Sensor for detection and degradation studies of ethyl violet dye. ACS Omega.

[23] Xie C., Niu Z., Kim D., Li M., Yang P. (2019). Surface and interface control in nanoparticle catalysis. Chem. Rev..

[24] Baek D.S., Joo S.H. (2022). Non-siliceous ordered mesoporous materials via nanocasting for small molecule conversion electrocatalysis. Bull. Korean Chem. Soc..

[25] Luo X., Morrin A., Killard A.J., Smyth M.R. (2006). Application of nanoparticles in electrochemical sensors and biosensors. Electroanal. Int. J. Devoted Fundam. Pract. Asp. Electroanal..

[26] Gao X., Lee J., White H.S. (1995). Natural convection at microelectrodes. Anal. Chem..

[27] Novev J.K., Compton R.G. (2018). Natural convection effects in electrochemical systems. Curr. Opin. Electrochem..

[28] Mateescu A., Wang Y., Dostalek J., Jonas U. (2012). Thin hydrogel films for optical biosensor applications. Membranes.

[29] Wang N., Wu X.S. (1997). Preparation and characterization of agarose hydrogel nanoparticles for protein and peptide drug delivery. Pharm. Dev..

[30] Fernández-Cossío S., León-Mateos A., Sampedro F.G., Oreja M.T.C. (2007). Biocompatibility of agarose gel as a dermal filler: Histologic evaluation of subcutaneous implants. Plast. Reconst. Surg..

[31] Zarrintaj P., Manouchehri S., Ahmadi Z., Saeb M.R., Urbanska A.M., Kaplan D.L., Mozafari M. (2018). Agarose-based biomaterials for tissue engineering. Carbohydr. Polym..

[32] Hayashi A., Kanzaki T. (1987). Swelling of agarose gel and its related changes. Food Hydrocoll..

[33] Rochas C., Hecht A.M., Geissler E. (1996). Swelling properties of agarose gels. J. Chim. Phys..

[34] Wang S.F., Chen T., Zhang Z.L., Shen X.C., Lu Z.X., Pang D.W., Wong K.Y. (2005). Direct electrochemistry and electrocatalysis of heme proteins entrapped in agarose hydrogel films in room-temperature ionic liquids. Langmuir.

[35] Kim B.K., Park K. (2022). Mass Transport Properties and Influence of Natural Convection for Voltammetry at the Agarose Hydrogel Interface. J. Electrochem. Sci. Technol..

[36] Han J., Jang S., Kim B.K., Park K. (2023). Electrochemical study of agarose hydrogels for natural convection on macroelectrodes and ultramicroelectrodes. J. Anal. Sci. Technol..

[37] Ko S. (2022). Multifunctional surface coating using chitosan and its chemical functionalization. Bull. Korean Chem. Soc..

[38] Reddy S.M., Sette G., Phan Q. (2011). Electrochemical probing of selective haemoglobin binding in hydrogel-based molecularly imprinted polymers. Electrochim. Acta.

[39] Maity S., Parshi N., Prodhan C., Chaudhuri K., Ganguly J. (2018). Characterization of a fluorescent hydrogel synthesized using chitosan, polyvinyl alcohol and 9-anthraldehyde for the selective detection and discrimination of trace Fe^3+^ and Fe^2+^ in water for live-cell imaging. Carbohydr. Polym..

[40] Shi Y., Peng L., Yu G. (2015). Nanostructured conducting polymer hydrogels for energy storage applications. Nanoscale.

[41] Zhang W., Feng P., Chen J., Zhao B. (2018). Flexible energy storage systems based on electrically conductive hydrogels. Prog. Polym. Sci..

[42] Ismail Y.A., Martínez J.G., Al Harrasi A.S., Kim S.J., Otero T.F. (2011). Sensing characteristics of a conducting polymer/hydrogel hybrid microfiber artificial muscle. Sens. Actuators B Chem..

[43] Zhao Z., Fang R., Rong Q., Liu M. (2017). Bioinspired nanocomposite hydrogels with highly ordered structures. Adv. Mater..

[44] Muya F.N., Baker P.G., Iwuoha E.I. (2020). Polysulfone hydrogel nanocomposite alkaline phosphatase biosensor for the detection of vanadium. Electrocatalysis.

[45] Zeng X., Wei W., Li X., Zeng J., Wu L. (2007). Direct electrochemistry and electrocatalysis of hemoglobin entrapped in semi-interpenetrating polymer network hydrogel based on polyacrylamide and chitosan. Bioelectrochemistry.

[46] Dewan M., Adhikari A., Dutta K., Chattopadhyay D. (2023). Impact of Poly (Vinyl Alcohol) on The Thermogelation Property and Drug Release Profile of Ophthalmic Formulations Based on Poloxamer 407. ChemistrySelect.

[47] Ganguly S., Margel S. (2022). 3D printed magnetic polymer composite hydrogels for hyperthermia and magnetic field driven structural manipulation. Prog. Polym. Sci..

[48] Garai S., Chatterjee D., Mondal B. (2023). Effect of rotation and cross thermal buoyancy on the nanofluidic transport around a circular cylinder. Phys. Fluids.

[49] Haleem A., Pan J.M., Shah A., Hussain H., He W.D. (2023). A systematic review on new advancement and assessment of emerging polymeric cryogels for environmental sustainability and energy production. Sep. Purif. Technol..

[50] Kleijn S.E., Serrano-Bou B., Yanson A.I., Koper M.T. (2013). Influence of hydrazine-induced aggregation on the electrochemical detection of platinum nanoparticles. Langmuir.

[51] Maaloum M., Pernodet N., Tinland B. (1998). Agarose gel structure using atomic force microscopy: Gel concentration and ionic strength effects. Electrophoresis.

[52] Kwon S.J., Zhou H., Fan F.R.F., Vorobyev V., Zhang B., Bard A.J. (2011). Stochastic electrochemistry with electrocatalytic nanoparticles at inert ultramicroelectrodes—Theory and experiments. Phys. Chem. Chem. Phys..

[53] Bard A.J., Faulkner L.R. (2001). Electrochemical Methods, Fundamentals and Applications.

[54] Johnson E.M., Berk D.A., Jain R.K., Deen W.M. (1996). Hindered diffusion in agarose gels: Test of effective medium model. Biophys..

[55] Stellwagen N.C. (1992). Agarose gel pore radii are not dependent on the casting buffer. Electrophoresis.

[56] Narayanan J., Xiong J.Y., Liu X.Y. (2006). Determination of agarose gel pore size: Absorbance measurements vis a vis other techniques. J. Phys. Conf. Ser..

[57] Cuccia N.L., Pothineni S., Wu B., Méndez Harper J., Burton J.C. (2020). Pore-size dependence and slow relaxation of hydrogel friction on smooth surfaces. Proc. Natl. Acad. Sci. USA.

[58] Jayawardena I., Turunen P., Garms B.C., Rowan A., Corrie S., Grøndahl L. (2023). Evaluation of techniques used for visualisation of hydrogel morphology and determination of pore size distributions. Mater. Adv..

[59] Sathawong S., Sridach W., Techato K.A. (2018). Recovery of Kraft lignin from OPEFB and using for lignin–agarose hydrogel. J. Polym. Environ..

[60] Mehta G.K., Kondaveeti S., Siddhanta A.K. (2011). Facile synthesis of agarose-l-phenylalanine ester hydrogels. Polym. Chem..

[61] Xiao X., Fan F.R.F., Zhou J., Bard A.J. (2008). Current transients in single nanoparticle collision events. J. Am. Chem. Soc..

